# Comparison of Pre-existing Mood Disorders and Chronic Kidney Disease as Predictors of Ambulatory Status After Major Limb Amputation

**DOI:** 10.7759/cureus.39215

**Published:** 2023-05-19

**Authors:** Natalie Chao, Maria Som, Eyerusalem Workneh, Allison Karwoski, Eleanor Dunlap, Suzanna Fitzpatrick, Khanjan Nagarsheth

**Affiliations:** 1 Vascular Surgery, University of Maryland School of Medicine, Baltimore, USA; 2 Vascular Surgery, University of Maryland Medical Center, Baltimore, USA

**Keywords:** lower limb amputation, parent-provider communication, adequate follow-up, psychosocial support, social support network, chronic kidney disease (ckd), psychiatric comorbidities, post-operative mobility, major limb amputation

## Abstract

Objective

We aim to compare the effects of pre-existing mood disorders and chronic kidney disease (CKD) on ambulation outcomes for patients who have undergone major lower extremity amputation (MLEA) while also stratifying by the presence of social factors.

Methods

We performed a retrospective chart review of 700 patients admitted from 2014 to 2022 who underwent MLEA. We performed Chi-square tests and binomial logistic regression with p < 0.05 as our significance level.

Results

Mood disorder patients have higher rates of independent ambulation if they have familial support (p = 0.022), a listed primary care provider (PCP; p = 0.013), a six-month follow-up (p < 0.001), or a one-year follow-up (p < 0.001). Patients with a history of mood disorder have significantly decreased odds of prosthesis usage (OR: 0.58, 95% CI: 0.40-0.86) but have higher rates of prosthesis usage if they have familial support (p = 0.002), a PCP listed (p = 0.005), a six-month follow-up (p < 0.001), or a one-year follow-up (p < 0.001). CKD patients have significantly decreased odds of eventual independent ambulation (OR: 0.69, 95% CI: 0.49-0.97) but have significantly increased rates of independent ambulation if they have familial support (p =0.041) and six-month (p < 0.001) or one-year follow-up (p < 0.001). CKD patients only have significant changes in prosthesis usage with a six-month (p < 0.001) or one-year follow-up (p < 0.001).

Conclusions

Pre-existing CKD and mood disorders are associated with decreased odds of independent ambulation and prosthesis usage, respectively. Social factors such as family support, a listed PCP, and timely follow-up are associated with markedly improved ambulatory outcomes for MLEA patients with mood disorders and CKD, with significantly improved prosthesis usage outcomes in only the mood disorder population.

## Introduction

Major lower extremity amputation (MLEA) is a life-altering procedure that can result in reduced mobility and impairment in performing activities of daily living. Less than 50% of patients are ambulatory one-year post-procedure [1], and even fewer ever ambulate with a prosthesis [2,3]. Being able to walk independently with a prosthesis has been cited as a key factor for enhanced quality of life (QoL) after amputation [4], and patients struggling to ambulate post-amputation may also experience negative body image [5]. These stressors can lead to MLEA patients developing depressive symptoms at 3-51% greater risk than the general population [6,7].

A pre-existing history of psychiatric illness has varied and established predictive value for ambulation. A retrospective review of 154 patients by Nehler et al. found that while patients with a history of psychiatric illness had no change in ambulation compared to patients without, they did have a significant reduction in prosthesis usage [2]. Another study by Yilmaz et al. of 135 patients noted that MLEA patients with lower postoperative functional status had higher levels of anxiety and depression [8]. Finally, Parker et al. assessed 52 MLEA patients for ambulation capacity and found that pre-existing depression was the only factor related to self-reported decreased independent ambulation [9]. Independent ambulation is also influenced by physical comorbidities. Lower limb amputation often results from diabetes mellitus (DM), peripheral arterial disease (PAD), or renal failure, which are also associated with lower rates of ambulation [10,11]. End-stage renal disease (ESRD) has been identified as a significant predictor of MLEA, and even less severe levels of renal disease can negatively affect limb salvage and increase rates of amputation [12,13]. A cross-sectional analysis by Luders et al. showed that patients with chronic kidney disease (CKD) had a 1.8-fold higher amputation rate, which increased stepwise as renal failure progressed [14]. While both CKD and psychiatric illness have been individually shown to relate to amputation outcomes, their effects on ambulation have not yet been analyzed and compared.

We aim to investigate how the documented history of psychiatric illness with depressive symptoms affects ambulation outcomes differently than pre-existing CKD in MLEA patients at an urban, large-volume tertiary medical center. We also examined how medical and emotional support in the form of patient-reported social support, an identifiable PCP, and timely follow-up affect ambulation outcomes in both the mood disorder and CKD patient populations. We hypothesize that pre-existing mood disorders and CKD both have a negative impact on mobility, but that patients with social support, a PCP listed, or regular follow-up will correlate with higher rates of independent ambulation and prosthesis usage.

## Materials and methods

This retrospective review was conducted with IRB approval (HP-00085462_4) through a chart review of MLEAs.

Inclusion and exclusion criteria

**Figure 1:**
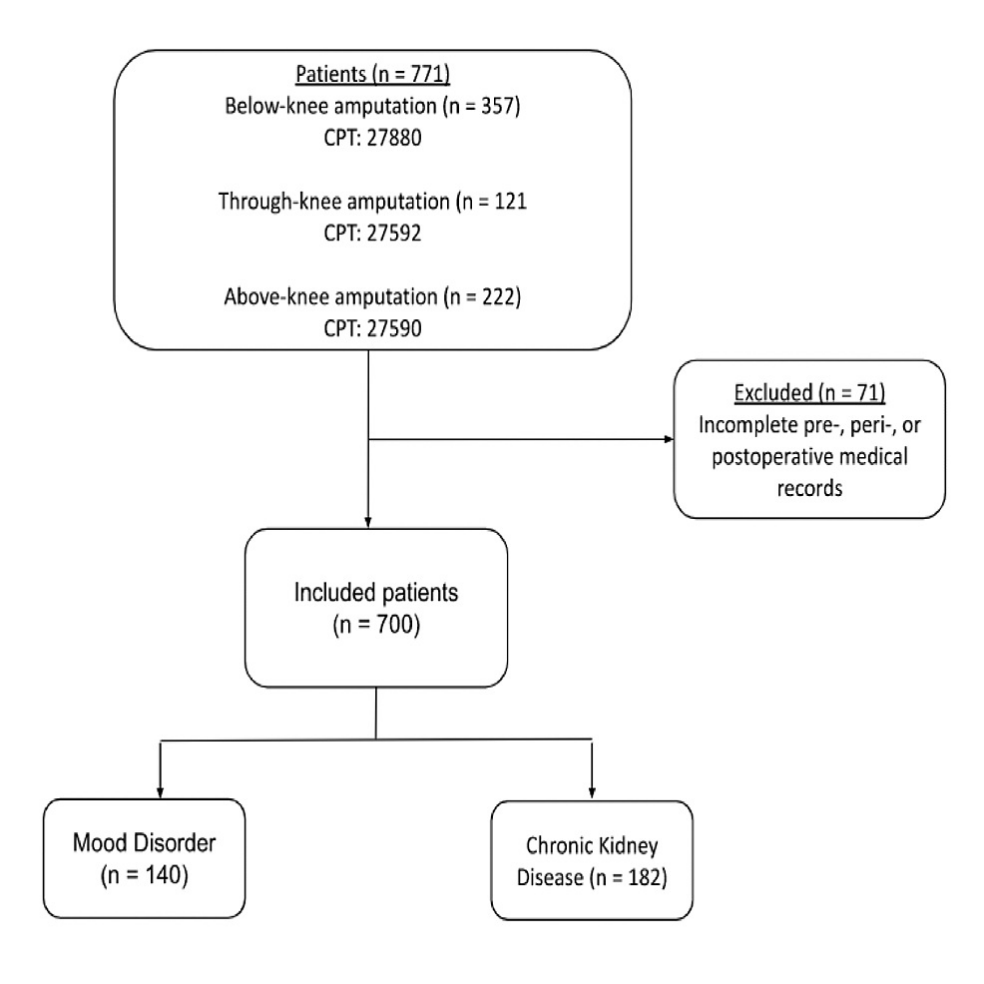
Flow diagram of selection for current retrospective study

Inclusion and exclusion criteria are outlined in Figure 1. We included initial below-knee amputations (BKA), through-knee amputations (TKA), and above-knee amputations (AKAs) performed at the institution. We did not include amputation revisions as a separate amputation in the population. Patients were included if they were ≥18 years old, had received MLEA, and had complete pre-, peri-, and post-operative records in our electronic medical record (EMR). We extracted data from Epic records of patients with CPT codes related to major amputation or disarticulation (BKA: 27880, TKA: 27592, AKA: 27590). We excluded patients with incomplete pre-, peri-, and/or postoperative records or whose charts were not detailed enough for reasonable interpretation of omitted data points (i.e., patients with clear lung findings and/or no mention of respiratory distress or respiratory condition would be noted as not having chronic obstructive pulmonary disease).

We included patients with a pre-existing diagnosis of bipolar disorder or major depressive disorder formally documented in their medical history by a clinician. We focused on these two diagnoses because they were the most common types of mood disorders in our patient population; we did not identify patients with prior diagnoses of other mood disorders such as dysthymic disorder, mood disorder due to a medical condition, or substance-induced mood disorder in our population. We included patients with all levels of CKD (stages 1-5). Patients were identified as having a diagnosis of mood disorder or CKD if it was noted in their documented medical history in EMR by a clinician or if they had a related ICD-10 code preceding the date of MLEA. We compared the CKD patient population and the mood disorder patient population as homogenous groups and did not stratify their results by the severity of the CKD stage or a specific type of mood disorder. Social support was self-reported by patients in their EMR and categorized by their emergency contact. Patients had the option of classifying their emergency contact as "family" or "other," with more specific identifiers such as "mother" and "father" being classified as familial and "friend" being classified as "other." Patients with either "none" or "unknown" for their emergency contact were classified as having "none" for social support. Provider support was defined by the presence of a listed primary provider (PCP) in the EMR as well as the presence of follow-ups at six months and/or one year postoperatively. The PCP listed was self-reported by patients, and follow-up status was documented in the chart via visit notes by clinicians.

Data obtained from the EMR review included demographics (age, sex, race, ethnicity), social support (familial, other, none), PCP-listed medical comorbidities, tobacco use, intravenous drug use (IVDU), or other opiate addiction. The preoperative variables reviewed were the indication for amputation and preoperative serum creatinine levels. Operative variables included the date of procedure, level, method (staged vs. one-stage), operative time, and operating service. Finally, postoperative variables were the length of stay (LOS), medical complications, mortality, death date (if applicable), six-month- and one-year follow-up, independent ambulation status, and prosthesis usage. Postoperative ambulatory status was defined as "the ability to walk, with or without the aid of assistive devices, safely and sufficiently to carry out mobility-related activities of daily living" after one year postoperative and was documented in the patient chart by physical therapists, physical medicine and rehabilitation physicians, and/or by the operating surgeon [15]. Patients were classified as "yes" for the ability to independently ambulate if it was explicitly stated in the patient chart or if the language used in the chart was strongly suggestive of independent ambulation (i.e., the patient reports being able to walk with no human assistance). Patients were classified as "no" for the ability to independently ambulate if it was not explicitly stated in the chart or language was not used that was strongly suggestive of independent ambulation. Operative evaluation factors such as staged vs. one-stage amputation, amputation level, and amputation technique were decided by the operating surgeon and documented in the operative note.

Statistical analysis

Descriptive statistics are represented as numbers with percentages (frequency), and continuous variables are represented as mean ± standard deviation. Chi-square tests of association between independent pre-, peri-, and postoperative variables and independent ambulation or prosthesis usage were used to assess their statistical significance at the level of p < 0.05. Independent t-tests were used for comparing the continuous variable of LOS and were assessed at p < 0.05 significance. Associations of social support, PCP listing, and regular follow-ups with ambulatory outcomes in the mood disorder and CKD populations were also analyzed via Chi-square tests of the association at a level of p < 0.05.

Statistically significant independent variables from this univariate analysis were then entered into stepwise binomial logistic regressions in order to assess their individual impacts on ambulatory outcome odds ratios at the level of p < 0.05. Univariate and multivariate analyses were performed using Jamovi version 2.2 (The Jamovi Project, Sydney, Australia) and R Statistical Software (v.4.1.2; R Core Team 2021, RStudio, PBC, Boston, MA).

## Results

Demographics, complications, and comorbidities

Demographics are reported in Table 1. Between March 2014 and August 2022, 700 MLEAs were performed (51.0% BKAs, 17.3% TKAs, and 31.7% AKAs) among patients (47.7% black; 68.9% male; mean age 57.6 ± 15) for infection (n = 412, 58.9%), ischemia (n = 163, 23.2%), trauma (n = 102, 14.6%), or other causes including compartment syndrome and sarcoma (n = 23, 3.29%). 20% (n = 140) of patients had a history of mood disorder as defined by bipolar disorder (n = 33, 15.3%) or major depressive disorder (n = 107, 49.5%). 26.5% (n = 185) of patients have CKD at any stage.

**Table 1:** Demographics and presentation Data are presented as mean (standard deviation) or number (%). PAD: peripheral arterial disease; BKA: below-knee amputation; TKA: through-knee amputation; AKA: above-knee amputation; CKD: chronic kidney disease; eGFR: estimated glomerular filtration rate mL/min; ESRD: end-stage renal disease.

Variable	All patients (n = 700)
Age, years	57.6 (±15)
Male sex	482 (69)
Race	
Black	334 (48)
White	328 (47)
Other	38 (5)
Indication	
Infectious	412 (59)
Ischemia	163 (23)
Trauma	102 (15)
Other	23 (3)
Initial level	
BKA	357 (51)
TKA	121 (17)
AKA	222 (32)
Mood disorder	
None	574 (82)
Major depressive disorder	93 (13)
Bipolar disorder	19 (3)
Both/other unspecified	14 (2)
CKD (eGFR range)	
None	515 (74)
Stage 1 (eGFR: >90 mL/min)	10 (1)
Stage 2 (eGFR: 60–89 mL/min)	20 (3)
Stage 3a (eGFR: 45–59 mL/min)	23 (3)
Stage 3b (eGFR: 30–44 mL/min)	21 (3)
Stage 4 (eGFR: 15–29 mL/min)	11 (2)
Stage 5/ESRD (eGFR: <15 mL/min)	100 (14)
Mortality	152 (22)

Complications and comorbidities are reported in Table 2. Patients experienced significantly lower rates of independent postoperative ambulation if they experienced sepsis (p < 0.001), myocardial infarction (MI) (p = 0.005), or renal complications (p = 0.043). Patients also experienced lower rates of prosthesis usage if they experienced sepsis (p < 0.001), MI (p = 0.027), renal complications (p < 0.001), or urinary tract infection (p = 0.019).

**Table 2:** Comorbidities and complications stratified by independent ambulation and prosthesis usage status Data are presented as mean (standard deviation) or number (%). *Statistically significant. DM: diabetes mellitus; COPD: chronic obstructive pulmonary disease; PVD: peripheral venous disease; CAD: coronary artery disease; CKD: chronic kidney disease; IVDU: intravenous drug usage; PE: pulmonary embolism; UTI: urinary tract infection; CNS-CVA: central nervous system-cerebrovascular accident; MI: myocardial infarction; DVT: deep venous thrombosis.

Variable	Ambulatory (n = 343)	Non-ambulatory (n = 357)	P-value	Prosthesis usage (n = 264)	No prosthesis (n = 436)	P-value
Comorbidity						
Smoker			<0.001*			<0.001*
Never	121 (35)	98 (27)		71 (27)	128 (29)	
Current	86 (25)	113 (32)		98 (37)	155 (36)	
Former	131 (38)	122 (34)		94 (35)	125 (29)	
Unknown	5 (1)	24 (7)		1 (0.4)	28 (6)	
DM	180 (52)	188 (53)	0.961	139 (53)	229 (53)	0.974
COPD	35 (10)	43 (12)	0.439	30 (11)	48 (11)	0.885
Hypertension	234 (68)	241 (68)	0.840	179 (68)	296 (68)	0.981
Hyperlipidemia	139 (41)	141 (39)	0.781	111 (42)	169 (39)	0.390
PVD	145 (42)	171 (48)	0.135	111 (42)	205 (47)	0.200*
CAD	77 (22)	114 (32)	0.005*	53 (20)	138 (32)	<0.001*
Heart failure	44 (13)	97 (27)	<0.001*	37 (14)	104 (24)	0.002*
CKD	78 (23)	107 (30)	0.030*	68 (26)	117 (27)	0.754
Dialysis	38 (11)	58 (16)	0.047*	36 (14)	60 (14)	0.963
Malignancy	37 (11)	32 (9)	0.418	27 (10)	42 (10)	0.798
Heme disorder	136 (40)	149 (42)	0.574	108 (41)	177 (41)	0.935
Mood disorder	71 (21)	55 (15)	0.068	61 (23)	65 (15)	0.006*
Osteomyelitis	80 (23)	65 (18)	0.095	64 (24)	81 (19)	0.073
IVDU	71 (21)	62 (17)	0.180	52 (20)	81 (19)	0.266
Periodontal disease	4 (1)	4 (1)	0.955	3 (1)	5 (1)	0.990
Complications						
Sepsis	54 (16)	98 (27)	<0.001*	30 (13)	121 (28)	<0.001*
Pneumonia	9 (3)	13 (4)	0.460	9 (3)	13 (3)	0.703
PE	11 (3)	13 (4)	0.752	10 (4)	14 (3)	0.682
Renal	65 (19)	97 (27)	0.045*	39 (15)	123 (28)	<0.001*
UTI	22 (6)	31 (9)	0.261	12 (5)	41 (9)	0.019*
CNS-CVA	7 (2)	18 (5)	0.111	6 (2)	19 (4)	0.417
MI	9 (3)	26 (7)	0.005*	7 (3)	28 (6)	0.027*
DVT	22 (6)	19 (5)	0.538	20 (8)	21 (5)	0.131

Patients had lower rates of independent ambulation if they were former smokers (p < 0.001) or current smokers (p < 0.001) and if they had a history of coronary artery disease (p = 0.005), heart failure (p < 0.001), dialysis (p = 0.047), or CKD (p = 0.030). Patients also had lower rates of prosthesis usage if they were former smokers (p < 0.001) or current smokers (p < 0.001) and if they had a history of coronary artery disease (p < 0.001) or heart failure (p = 0.002).

Mortality was significantly increased in the CKD population (35.7%, p < 0.001) but was not significant in the mood disorder population (p = 0.359).

Stratification by level of initial amputation and LOS

Patients in the CKD population had significantly different rates of independent ambulation (p < 0.001) among BKA (n = 59, 55.7%), TKA (n = 7, 25.9%), and AKA (n = 12, 23.1%). Patients with a history of mood disorder did not have significantly different rates of independent ambulation by level (p = 0.214) but had significantly different rates of prosthesis usage (p = 0.044) among BKA (n = 37, 56.9%), TKA (n = 4, 23.5%), and AKA (n = 20, 45.5%).

Patients with mood disorders had significantly longer mean LOS with greater variance (23.4 ± 30.7 days vs. 18.9 ± 18.0 days, p = 0.028). Patients with CKD did not have a significant difference in LOS (21.0 ± 21.8 days vs. 19.2 ± 20.7 days, p = 0.33).

Social support, provider support, and follow-up

Psychosocial factors impacting ambulatory status are detailed in Tables 3-4. Mood disorder patients who had familial support (p = 0.022), a PCP listed (p = 0.013), six-month (p < 0.001), or one-year (p < 0.001) follow-up had higher rates of independent ambulation than mood disorder patients without these factors. Mood disorder patients also had higher rates of prosthesis usage if they had familial support (p = 0.002), a PCP listed (p = 0.005), a six-month follow-up (p < 0.001), or a one-year follow-up (p < 0.001). CKD patients only had a statistically significant change in rates of independent ambulation if they had familial support (p = 0.041), six-month (p < 0.001), or one-year (p < 0.001) follow-up. CKD patients only had a significant increase in prosthesis usage with six-month (p < 0.001) and one-year (p < 0.001) follow-ups but did not have a significant increase with social support (p = 0.725) or a listed PCP (0.201).

**Table 3:** Social support, listed primary care provider, and follow-up stratified by ambulatory status for mood disorder patients Data are presented as mean (standard deviation) or number (%). *Statistically significant; PCP: primary care provider.

Variable	Ambulatory (n = 71)	Non-ambulatory (n = 55)	P-value	Prosthesis usage (n = 61)	No prosthesis (n = 65)	P-value
Social support						
Family	65 (92)	47 (85)	0.022*	56 (92)	56 (86)	0.002*
Other	6 (9)	5 (9)	0.547	5 (8)	6 (9)	0.785
None	0 (0)	3 (5)	0.149	0 (0)	3 (5)	0.259
Follow-up						
6-month	68 (96)	31 (56)	<0.001*	59 (97)	40 (62)	<0.001*
1-year	49 (69)	10 (18)	<0.001*	46 (75)	13 (20)	<0.001*
PCP listed						
Present	63 (89)	38 (69)	0.013*	54 (89)	47 (72)	0.005*
None	8 (11)	17 (31)	0.344	8 (13)	17 (26)	0.685

**Table 4:** Social support, primary care provider, and follow-up stratified by ambulatory status for chronic kidney disease population Data are presented as mean (standard deviation) or number (%). *Statistically significant. PCP: primary care provider; CKD: chronic kidney disease.

Variable	Ambulatory (n = 78)	Non-ambulatory (n = 107)	P-value	Prosthesis usage (n = 68)	No prosthesis (n = 117)	P-value
Social support						
Family	72 (92)	101 (94)	0.041*	62 (91)	111 (95)	0.725
Other	5 (6)	5 (5)	0.451	5 (7)	5 (4)	0.955
None	1 (1)	1 (0.9)	0.683	1 (1)	1 (0.9)	0.421
Follow-up						
6-month	74 (95)	48 (45)	<0.001*	66 (97)	56 (48)	<0.001*
1-year	49 (63)	15 (14)	<0.001*	47 (69)	17 (15)	<0.001*
PCP listed						
Present	71 (91)	97 (91)	0.931	62 (91)	106 (91)	0.201
None	7 (9)	10 (9)	0.946	6 (9)	11 (9)	0.287

Binomial regression analyses of variable effects on ambulatory status are detailed in Table 5. Heart failure (p < 0.001), CKD (p = 0.032), being a current (p = 0.014) or unknown (p < 0.001) smoker, CAD (p = 0.005), sepsis (p = 0.001), MI (p = 0.034), TKA (p < 0.001), and AKA (p < 0.001) all significantly decreased the odds of independent ambulation. Listed PCP (p = 0.023), six-month follow-up (p < 0.001), and one-year follow-up (p < 0.001) significantly increased the odds of independent ambulation. Heart failure (p = 0.002), mood disorder (p = 0.007), unknown smoking status (p = 0.003), CAD (p < 0.001), sepsis (p < 0.001), renal complications (p = 0.017), TKA (p < 0.001), and AKA (p < 0.001) significantly decreased odds of prosthesis usage, while six-month (p < 0.001) and one-year follow-up (p < 0.001) significantly increased odds of prosthesis usage.

**Table 5:** Regression analysis of the variable effect on independent ambulation and prosthesis usage *Statistically significant. TKA: through-knee amputation; AKA: above-knee amputation; PCP: primary care provider.

	Independent ambulation		Prosthesis usage	
Variable	OR (95% CI)	P-value	OR (95% CI)	P-value
Mood disorder	0.70 (0.47–1.03)	0.072	0.58 (0.40–0.86)	0.007*
CKD	0.69 (0.49–0.97)	0.032*	0.95 (0.67–1.35)	0.776
Comorbidity				
Heart failure	0.394 (0.27–0.58)	<0.001*	0.52 (0.35–0.79)	0.002*
Smoker				
Current	0.62 (0.42–0.91)	0.014*	0.74 (0.50–1.09)	0.131
Former	0.87 (0.60–1.25)	0.451	0.84 (0.58–1.22)	0.356
Unknown	0.17 (0.06–0.46)	<0.001*	0.05 (0.006–0.36)	0.003*
CAD	0.62 (0.44–0.87)	0.005*	0.54 (0.38–0.78)	<0.001*
Dialysis	0.73 (0.46–1.15)	0.174	0.99 (0.63–1.54)	0.963
Complication				
Sepsis	0.51 (0.35–0.77)	0.001*	0.39 (0.24–0.61)	<0.001*
Renal	0.78 (0.54–1.15)	0.213	0.60 (0.40–0.91)	0.017*
MI	0.43 (0.19–0.94)	0.034*	0.56 (0.23–1.33)	0.187
Level of amputation				
TKA	0.28 (0.18–0.44)	<0.001*	0.31 (0.19–0.50)	<0.001*
AKA	0.34 (0.24–0.47)	<0.001*	0.38 (0.27–0.55)	<0.001*
PCP listed	1.54 (1.06–2.25)	0.023*	2.10 (1.38–2.10)	<0.001*
Follow-up				
6-month	13.5 (8.45–21.7)	<0.001*	11.7 (5.53–24.9)	<0.001*
1-year	6.53 (4.60–9.25)	<0.001*	6.17 (4.22–9.03)	<0.001*
Social support				
Family	1.60 (0.62–4.11)	0.332	1.64 (0.58–4.61)	0.348
Other	2.64 (0.91–7.66)	0.074	2.71 (0.87–8.45)	0.086

## Discussion

Our results highlight the significance of CKD and pre-existing mood disorders in the context of social support in predicting ambulatory status after MLEA. The presence of CKD significantly decreased the odds of independent ambulation, and a pre-existing history of mood disorders significantly decreased the odds of prosthesis usage. Patients with pre-existing mood disorders are associated with higher rates of independent ambulation and prosthesis usage if they have familial support, a listed PCP, and follow-up within six months and one-year post-procedure. While CKD patients with familial support and regular follow-up are associated with higher rates of independent ambulation, rates of prosthesis usage are only affected by the presence of six-month and one-year follow-ups.

These findings corroborate those of Yilmaz et al. and extend the conclusions from Parker et al. by indicating that mood disorders affect ambulation outside of self-reporting [8,9]. Ambulatory status was noted as independent only if explicitly reported in the chart by the medical team, and so these results demonstrate that mood disorder affects the external assessment of ambulation performance. The prosthesis usage results counter those of Larner et al., who found that depression was not a significant predictor of functional prosthesis use [16]. This discrepancy in findings may have been due to our larger sample size and our inclusion of bipolar disorder under the umbrella of mood disorders.

Patients with mood disorders did not have significant differences in independent ambulation rates across amputation levels but did have significant differences in prosthesis usage. Prosthesis fitting is dependent on the physical anatomy of the articulation point of the leg. Preservation of the body envelope via prosthesis has been shown to normalize body image in MLEA patients, which consequently increases self-esteem and motivation to pursue functional activities [17,18]. However, prostheses are expensive, and the fitting process can be lengthy, particularly for patients with higher MLEA levels, which could dissuade patients who already achieve satisfactory mobility with another assistive device. Familial support and PCP listing may therefore affect the odds of prosthesis usage rather than independent ambulation because patients can rely on their support network and better weather the cost and process of acquiring a prosthesis.

Furthermore, our study proves patients with formally diagnosed mood disorders can achieve high rates of independent ambulation with proper support. The ambulation rates in patients with mood disorders rival those of patients in the CKD population as well as those in patients without comorbidities, thus showing that pre-existing psychiatric illness should not be viewed as a harbinger of a poor outcome. This finding counters the conclusions by Norvell et al. that pre-existing depression decreases the chances of mobility success and raises expectations for QoL even with pre-existing psychiatric illness [19]. Pre-existing mood disorders should not preclude patients from limb salvage or from receiving a more distal amputation to preserve ambulatory potential, and preconceptions concerning the decreased ambulatory potential for mood disorder patients post-MLEA should be dismantled.

The relationship between pre-existing mood disorders and ambulation is important for clinicians to understand due to its effect on QoL. Pell et al. assessed a population of 149 amputation patients and found that mobility status was the single independent factor that significantly worsened QoL, with differences in feelings of isolation and distress losing significance when adjusted to functional status [20]. A study by Pedras et al. of 179 patients also found that patients with pre-amputation depressive symptoms had higher levels of depressive symptoms post-surgery, which contribute to poor psychological adjustment and QoL [21]. Furthermore, depressive symptoms can decrease the motivation to pursue functioning after amputation [22], and depressive symptoms may be more severe in patients with comorbid conditions who already experience greater physical stress [23]. Loss of pre-surgery functional status may also prevent patients from engaging in enjoyable activities or reaching out for social support that can improve post-procedure QoL [24].

Our findings indicate mental health support should be a priority for the surgical team and should be offered particularly to patients with comorbidities. Patients with a listed PCP and regular follow-ups have a closer relationship with their healthcare providers and have more opportunities to receive medical guidance. However, many patients anecdotally report a lack of support from their healthcare teams when facing limb amputation [25], and Darnall et al. reported that in a sample of 914 patients, 32.9% reported needing mental health services but not receiving them [23]. Since the surgical team is directly involved with the planning and execution of the amputation, they are positioned to offer resources, referrals, and support to patients. Patients who report having trust in and feeling supported by their surgical team are more likely to adhere to their suggestions and return for follow-up care, which in turn leads to more comprehensive and long-term care after MLEA. Ideally, the surgical team would be part of a multi-disciplinary team, including psychiatrists and outside organizations, in order to provide patients with access to a variety of mental health resources. For example, amputee support groups increase mobility success among patients who share the same goals of regaining functionality and using prostheses [26] and can serve as a bridge between provider and community support.

Additionally, the difference in significance between the mood disorder patient population and the CKD population suggests that patients with pre-existing psychiatric illnesses benefit more from psychosocial support than patients with only physical comorbidities. Positive ambulatory outcomes are associated with increased psychosocial support, which is paramount for patients with mood disorders who are more susceptible to depressive episodes. A study by Andrews et al. showed that while social factors impact both psychiatric and physical illness, a greater proportion of psychiatric aggravation could be directly attributed to family support [27]. Social support can alleviate the stress of adjusting to physical changes and lessen feelings of isolation from decreased mobility [28,29]. While social support is important for all patients, patients with psychiatric illnesses reap the utmost benefit, and support for them should be an even higher priority to encourage ambulation. The heightened importance of psychosocial factors in the mood disorder population when compared to their effect on CKD patients suggests that patient outreach should be tailored to maximize QoL on an individual-specific basis.

The strengths of our study lie in its large and diverse sample size, which assessed the novel variable of pre-existing mood disorder in the context of social support for ambulatory status. Yet, our study did have some limitations. We did not use rating scales for the assessment of mood disorders, so we were unable to validate the severity of the diagnosis. The severity of mood disorders may also affect the ability to ambulate or use prostheses, and future studies may benefit from rating scales and/or counting the presence and frequency of psychiatric hospitalizations in order to compare severity pre- and postoperatively. We stratified by the initial level of amputation, and further analysis using the final level of amputation is justified to determine how the residual level of amputation affects ambulation. Social support cannot be standardized, and definitions may vary for each patient. Patients who live with their emergency contact may feel more supported than patients who can only receive support via phone, which can result in patients with physical support at home experiencing less loneliness and consequently greater motivation to ambulate. Future studies using self-reported questionnaires would strengthen these correlations and stratify patients by the magnitude of social support they receive. Finally, a qualitative survey on QoL for patients with and without a history of mood disorders may be helpful to determine how they personally regard their mobility status after amputation.

## Conclusions

Pre-existing CKD and mood disorders are associated with decreased odds of reaching independent ambulation and prosthesis usage, respectively. Social factors such as familial support, a listed PCP, and timely follow-up are associated with improved independent ambulation outcomes for MLEA patients with mood disorders and CKD, but significantly improved prosthesis usage outcomes for only the mood disorder population. Future studies are warranted to examine which factors affect independent ambulation differently than prosthesis usage. In addition, further studies on insurance coverage and the socioeconomic status of patients able to obtain prostheses and independently ambulate would be valuable to determine the effect of patient financial situations on mobility outcomes.
